# Posteromedial Submeniscal Arthrotomy and Fixation with a Posteromedial Rim Plate in a Comminuted Medial Tibial Plateau Fracture

**DOI:** 10.1155/2023/3635067

**Published:** 2023-05-18

**Authors:** Nicolás Franulic, Tomás Pineda, José Laso, Diego Valiente, Nicolás Gaggero

**Affiliations:** ^1^Hospital del Trabajador ACHS, Santiago, Chile; ^2^Hospital Militar de Santiago, Santiago, Chile; ^3^Hospital El Carmen, Santiago, Chile; ^4^Hospital Barros Luco Trudeau, Santiago, Chile

## Abstract

Medial tibial plateau fractures generally present as simple metaphyseal fractures; however, certain cases may present as comminuted articular fractures. Medial and posteromedial anatomical plates have traditionally been used for their management; nevertheless, not all cases can be successfully managed using these implants. We present a comminuted posteromedial Schatzker type VI tibial plateau fracture case. Direct visualization and subsequent fixation using a posteromedial rim plate were achieved through a posteromedial approach and submeniscal arthrotomy. The adequate joint reduction and the obtained stability allowed satisfactory clinical and radiological outcomes. This variation of the classic posteromedial approach and the use of a posteromedial rim plate provide an alternative when facing comminuted medial tibial plateau fractures.

## 1. Introduction

Increasing knowledge about the natural history of tibial plateau fractures has led to an optimization of their study and management. As with any other articular fracture, anatomic restoration of joint congruence and alignment, along with stable fixation, is paramount to obtain satisfactory outcomes. Due to this, great interest has arisen in achieving an adequate reduction and fixation of the entire articular surface, a task difficult to accomplish with traditional anterolateral or anteromedial approaches and implants when it comes to posterolateral or posteromedial fragments.

To date, several reports of fixation with rim plates of the lateral and posterior columns have been published [1–8]. However, anteromedial rim plating remains of limited knowledge, and there are currently no reports in the literature of its use for the management of comminuted articular fractures of the posteromedial tibial plateau after visualization and reduction using a posteromedial submeniscal arthrotomy [7].

## 2. Case Presentation

**Figure 1:**
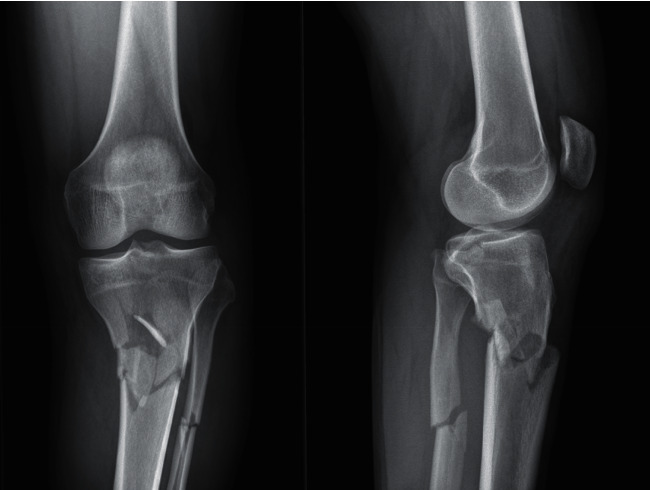
Pre-operative X-rays.

**Figure 2:**
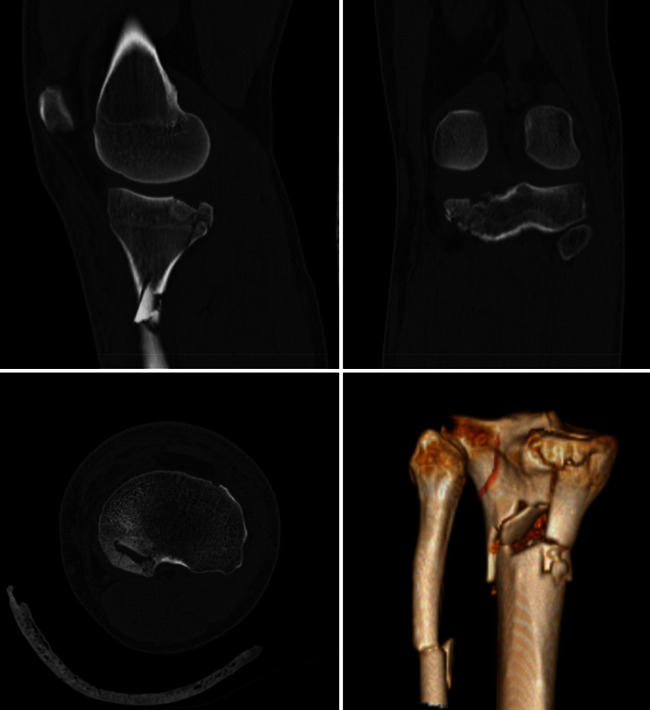
Pre-operative computed tomography and 3D reconstruction.

We present, with the consent of the patient, the case of a 46-year-old male patient with no relevant medical history who received a direct blow on his left knee by a container. He was transferred to the emergency department of our institution, where a Schatzker VI tibial plateau fracture was diagnosed (Figure 1). Reduction and transitory fixation with a left knee transarticular external fixator were performed. Definitive surgery was planned using anteroposterior and lateral left knee X-rays, computed tomography (CT) scan, and 3D knee reconstruction, showing posteromedial plateau comminution (Figure 2). Ten days after the accident, the external fixator was removed, and definitive open reduction and internal fixation were performed.

**Figure 3:**
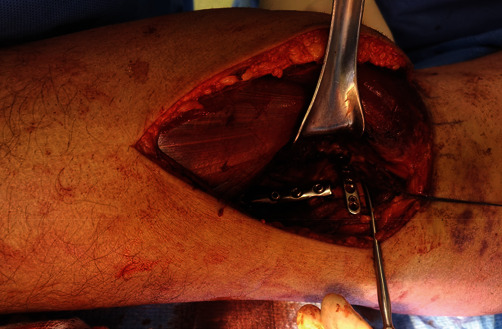
Direct visualization of the posteromedial articular surface of the medial tibial plateau by a submeniscal arthrotomy and fixation with a posteromedial rim plate.

With the patient in a prone position, an inverted L incision was made over the medial gastrocnemius muscle belly. The crural fascia was opened longitudinally, and the medial gastrocnemius was laterally retracted using Langenbeck and Richardson retractors. This maneuver protects the neurovascular bundle and exposes the popliteal muscle. A new inverted L-shaped incision was performed over the popliteus muscle, resembling the pronator quadratus elevation in the modified Henry's approach. Popliteus muscle belly subperiosteal elevation was then performed, exposing the posterior metaphyseal surface of the proximal tibia and allowing visualization of the fracture. The posterior cortical fragment was reduced and fixed with a six-hole third-tube locking plate. Using a cortical screw, direct fixation of the fragment was achieved. Then, a submeniscal posteromedial arthrotomy was performed, exposing the posteromedial comminuted tibial plateau. Traction sutures were passed at the posterior horn of the medial meniscus and were pulled proximally, allowing direct visualization of the articular surface and comminution. The main posteromedial comminuted fragments were elevated and temporarily fixed with Kirschner wires. Definitive fixation was achieved with a 2.7 mm precontoured mini fragment LCP Rim Plate (Compact foot 2.4–2.7 mm, DePuy Synthes; Figure 3).

Then, the patient was placed in supine position. Open reduction and internal fixation by classic anterolateral and anteromedial approaches with 4.5 mm plates were performed.

After surgery, a Robert Jones dressing was applied for 48 hours. Early isometric contraction of the muscles of both lower limbs with straight leg raise exercises for quadriceps strengthening were performed. Knee range of motion exercises were started the day after surgery to achieve a 90° flexion in the second post-operative week. The patient used two walking canes and remained non-weight-bearing for eight weeks after surgery. Following this time, progressive weight-bearing was encouraged.

**Figure 4:**
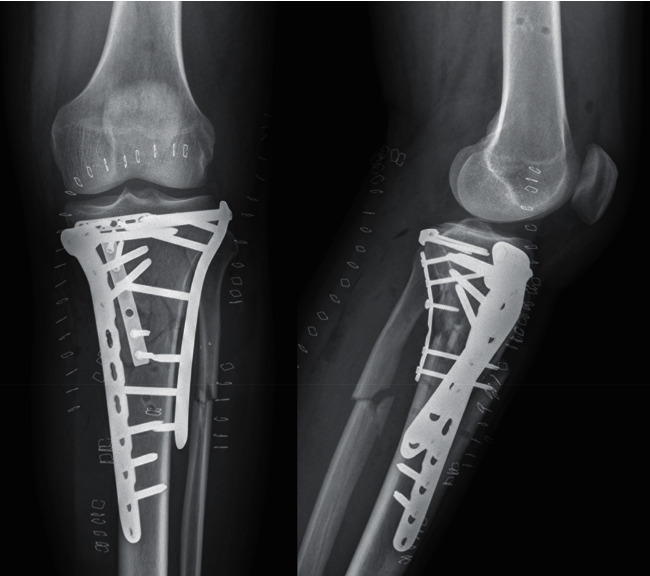
Post-operative X-rays.

**Figure 5:**
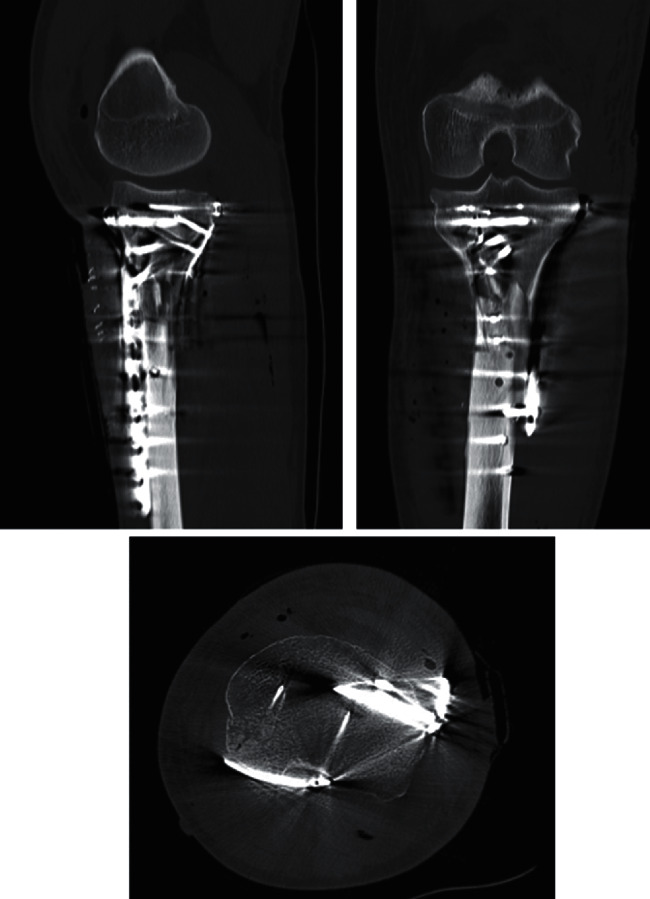
Post-operative computed tomography.

**Figure 6:**
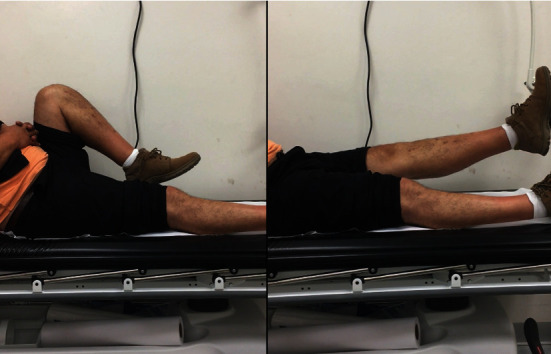
After 5 months, the patient presented full range of motion of the left knee and adequate muscle trophism.

Post-operative course was uneventful. Post-operative control X-rays and CT showed adequate reduction and fixation of the posteromedial comminution with the use of the rim plate (Figures 4 and 5). After five months, the patient presented full range of motion of the left knee (Figure 6), adequate muscle trophism, and achieved advanced radiological bone consolidation evidenced in CT. The patient returned to work six months after the accident. SF-36 score was measured at the ten months follow-up visit. The SF-36 physical score was 90.8, and the SF-36 mental score was 92.8.

## 3. Discussion

Medial tibial plateau fractures (Schatzker IV–VI) are considered highly demanding fractures despite being relatively uncommon [9, 10].

The surgical management of these fractures is a great challenge since multiple variables must be considered, such as subsidence, displacement, alignment, stability, and soft tissue status [11–13]. Among them, poor reduction and post-operative malalignment have been recognized as surgeon-dependent prognostic factors that negatively impact functional prognosis and can result in considerable morbidity and long-term disability [14, 15]. Therefore, an adequate fracture analysis and a pre-operative plan are necessary.

Currently, the most widely used classifications are based on the location of the fragments and allow inferring the mechanism of injury; however, the rim is not considered [11, 16–20].

Although the importance of containment of the articular ridge has yet to be fully elucidated, it has been reported that its inadequate reduction can lead to altered contact pressures on the articular surface and instability in flexion, leading to unacceptable results [21, 22]. Cuéllar et al. performed a biomechanical cadaveric study also evaluating fracture displacement under axial, torsional, varus, and valgus forces, finding significant fragment displacement under all evaluated forces and knee flexion angles except for 10 mm fragments under axial load and less than 30° flexion [23].

In recent years, the use of horizontal rim plates has been widely described for other columns [1–6]. However, despite the recent literature on the matter, its use in the posteromedial column isolated has barely been described [24]. To our knowledge, this is the first study to describe the association of a precontoured rim plate with a posteromedial submeniscal arthrotomy to allow direct articular surface observation, reduction, and fixation. In our case, we preferred to use a 2.7 mm locking plate (LCP Compact Foot, DePuy Synthes) for posteromedial rim plating, as described by Cho et al. for posterolateral columns with good results [3, 4]. These easily malleable plates allow us to precontour them with the proximal tibia shape. Another advantage is their stability since both 2.7 mm locked and cortical screws have been shown to provide adequate biomechanical stability. In an osteoporotic model of simple distal fibula fractures, three 2.7 mm screws demonstrated the same biomechanical stability as two 3.5 mm cortical screws [25].

On the other hand, these screws have a central diameter of 2.3 mm, whereas the 3.5 mm cortical screws have a central diameter of 2.4 mm, resulting in comparable rigidity and resistance to pull-out [26]. Finally, it should be considered that these plates can also be used in addition to conventional plates if it is necessary.

The current literature recommends the use of rim plates for the management of some lateral and posterior patterns; however, its use for the medial tibial plateau is less recognized. We present this case with a variation in the approach that reinforces the usefulness of the rim plates specifically in the posteromedial region with good results. Although studies with longer follow-up are still needed, we believe this is a useful tool in selected cases.

## Data Availability

Case report: data of the patients are in the clinical records of the hospital.
